# Supplementary material for: TRPC3 shapes the ER-mitochondria Ca2+ transfer characterizing tumour-promoting senescence
Source: Nat Commun. 2022 Feb 17;13:956. doi: 10.1038/s41467-022-28597-x (PMC8854551; doi:10.1038/s41467-022-28597-x)
Supplement: Supplementary file 1 — Supplementary Information [file 41467_2022_28597_MOESM1_ESM.pdf]

TRPC3 shapes the ER-mitochondria Ca^{2+} transfer characterizing tumour-promoting senescence

SUPPLEMENTARY INFORMATION

Supplementary Fig. 1

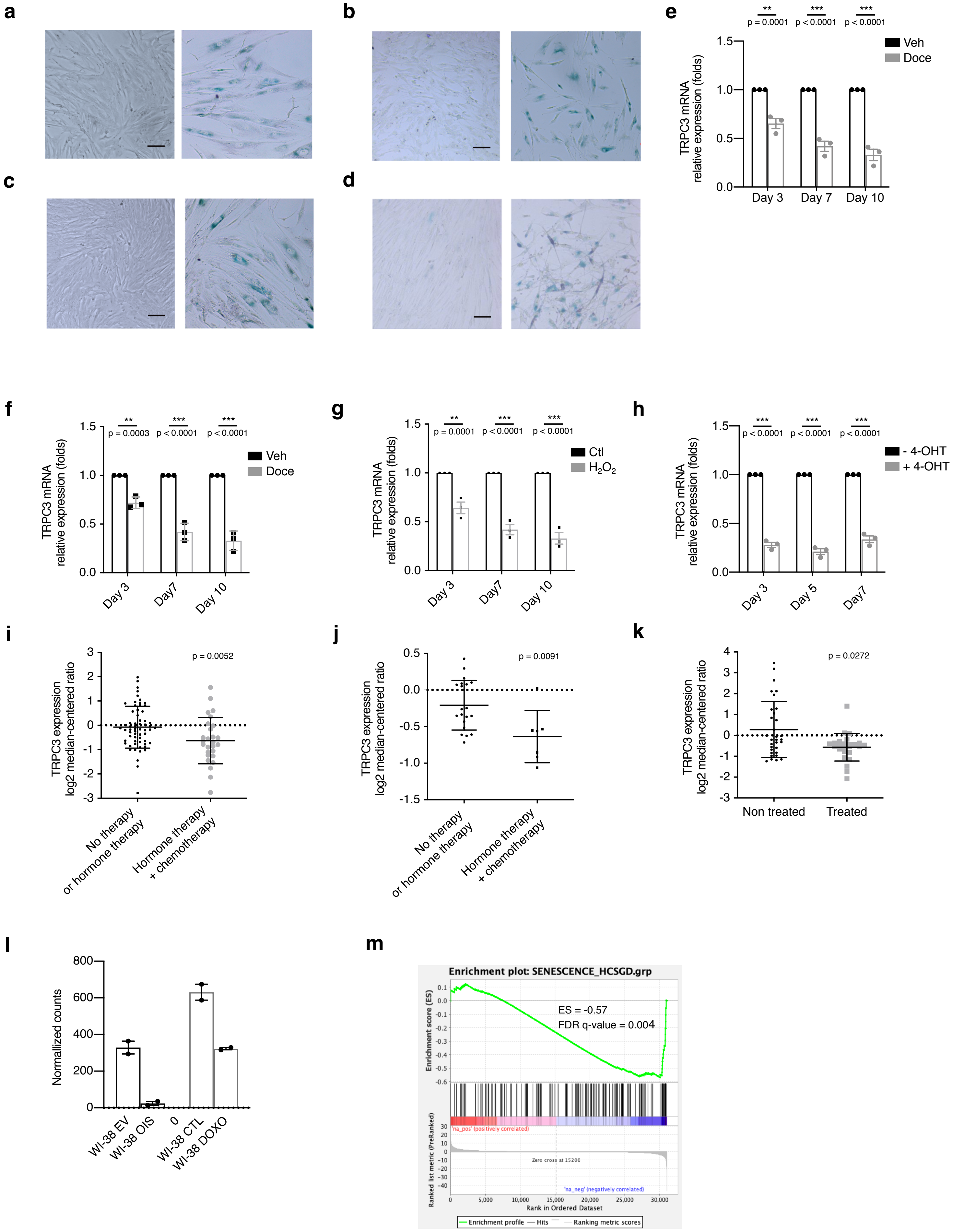

Supplementary Figure 1

(a-d) Validation of senescence models utilized for the readings presented in Fig.1A. The β-gal activity revealed with SAB-gal staining in HPrFs (A) and in prostate CAFs (B) both treated with either vehicle only (Veh) or 5 μM docetaxel, or in HPrFs (C) treated with 400 μM H_2O_2 vs. control (CTL: water) or in H-RAS^{G12V}-transduced MRC-5 fibroblasts (D) treated daily with 4-OHT (250 nM) to evoke oncogene-induced senescence. Staining was performed 10 days after senescence induction. Scale bar = 100 μm

(e-h) TRPC3 mRNA levels measured at different time points in the above described experimental models. Data are presented as mean ± S.E.M. (n = 3 biological independent experiments). $^{***}P<0.001$, $^{**}P<0.01$, $^{*}P<0.05$ (one-way ANOVA, Tukey's multiple comparisons test).

(i-j) TRPC3 expression, calculated from the log2 median centred ratios, in prostate cancer samples of patients undergoing or not chemo/radiotherapy treatment (n = 27 and n = 62, respectively). (I) Grasso prostate database. (J) Tamura prostate database (n = 7 and n = 20, respectively). Data are presented as mean ± S.D.

Data were derived from the Oncomine database. *P* values are presented on the plots (Student's t test, two-sided).

(k) TRPC3 expression, calculated from the log2 median centred ratios, in tumours of breast cancer patients undergoing or not chemo/radiotherapy treatment (n = 32 and n = 30, respectively). Data were derived from the Oncomine, Stickeler Breast dataset. *P*=0.0272 (Student's t test, two-sided).

(l) Log scale of normalized counts for TRPC3 obtained from dataset GSE130727 and showing TRPC3 expression in WI-38 fibroblasts in which senescence was induced either by OIS or CIS. Data are presented as mean ± S.E.M. (n = 2).

(m) GSEA of genes positively or negatively correlating with TRPC3 expression was performed on dataset GSE35988. Results for the "Senescence_UP" gene set (HCSGD) are shown.

Supplementary Fig. 2

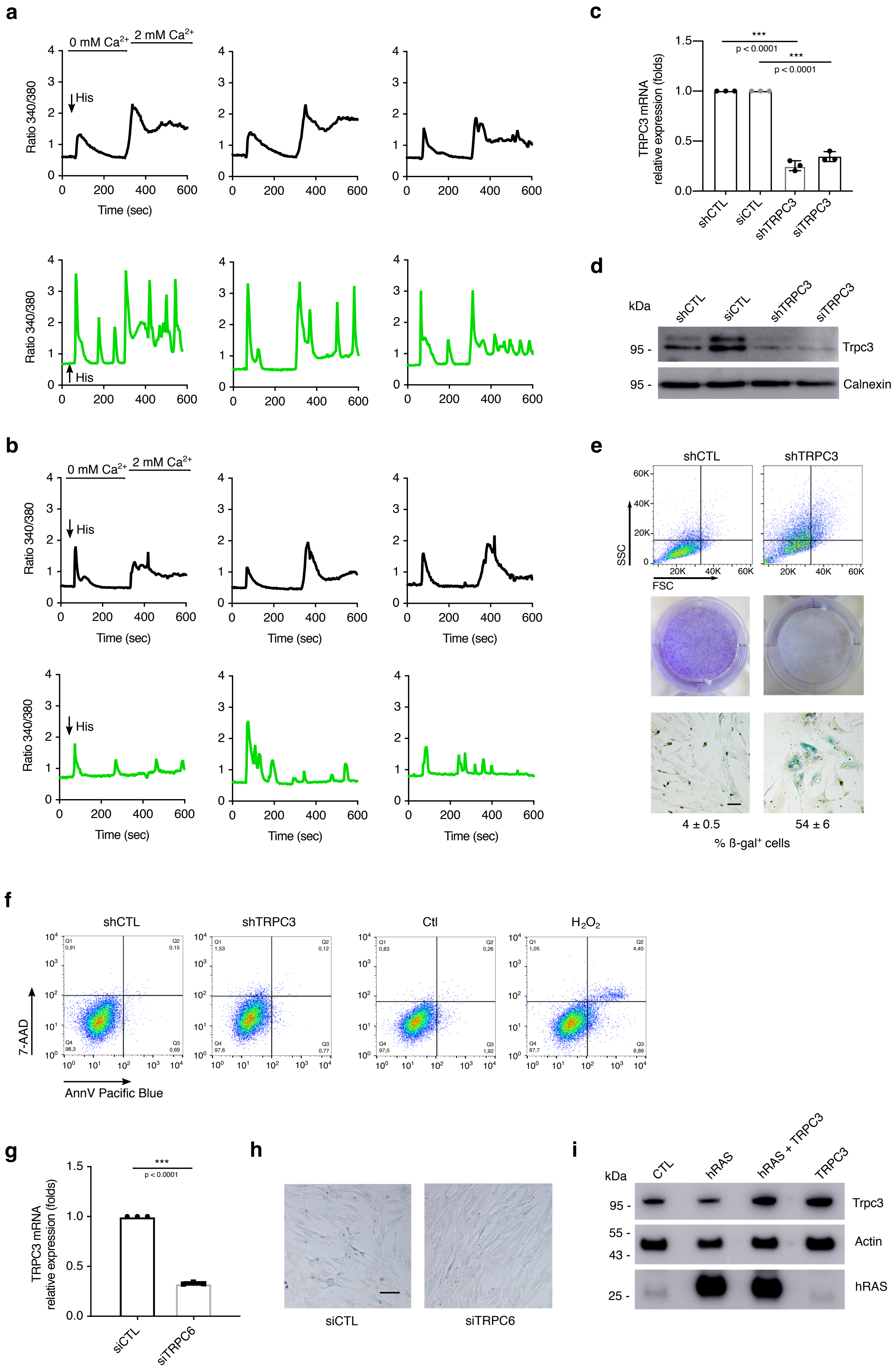

Supplementary Figure 2

(a-b) Cytosolic Ca^{2+} imaging (Fura-2) was performed on (A) CIS (Doce, 5 nM) or (B) OIS (4-OHT, 250 nM), 10 or 7 days after senescence induction respectively. Individual cell time-courses of fluorescence ratio with excitation wavelengths 340/380 nm. Note that DOX-treated cells are prone to cytosolic Ca^{2+} oscillations. His: histamine

(c-d) TRPC3 mRNA (C) and protein (D) levels 3 days after doxycycline treatment (500 nM) used to induce shRNA expression. Measurements are done in prostate fibroblasts transduced with either shTRPC3 or siTRPC3 and compared to their respective controls (shCTL or siCTL). Data are expressed as mean ± S.E.M. (n = 3). Western blot images are representative of three independent experiments. *** *P*<0.001 (Student's t test, two-sided).

(e) Effect of shRNA-mediated TRPC3 knockdown in HPrFs on: (Top) the cell structural parameters revealed with flow cytometry analysis – cell size (forward scatter; FSC) and internal complexity (side scattered, SSC), (Middle) cell growth reported by crystal violet staining and (Bottom) β-gal activity revealed with SA-β-gal staining (% of ß-galactosidase positive cells is shown, mean ± S.E.M). Readings were performed 10 days after TRPC3 knockdown. Scale bar = 150 μm.

(f) Left: flow cytometry analysis performed 10 days after doxycycline treatment of the shCTL- or shTRPC3-transduced prostate fibroblasts co-stained with Annexin-V Pacific Blue and 7-AAD. Right: as positive control for apoptosis induction the same analysis was performed on wild-type prostate fibroblasts treated with either water (Ctl) or 1 mM H_2O_2 for 24h. Images are representative of two independent experiments.

(g) TRPC6 mRNA levels in prostate fibroblasts 3 days after treatment with 10 nM of either siCTL or siTRPC6 oligonucleotides. Data are expressed as mean ± S.E.M. (n = 3). ****P*<0.001 (Student's t test, two-sided).

(h) SA-β-gal activity revealed with SA-β-gal staining in prostate fibroblasts treated with 10 nM of either siCTL or siTRPC6 oligonucleotides. Staining was performed 10 days after siRNA treatment. Images are representative of one out of three independent biological repeats. Scale bar = 100 μm.

(i) TRPC3 and hRAS protein expression upon induction with doxycycline or 4-OHT respectively. Western blot images are representative of three independent experiments.

Supplementary Fig. 3

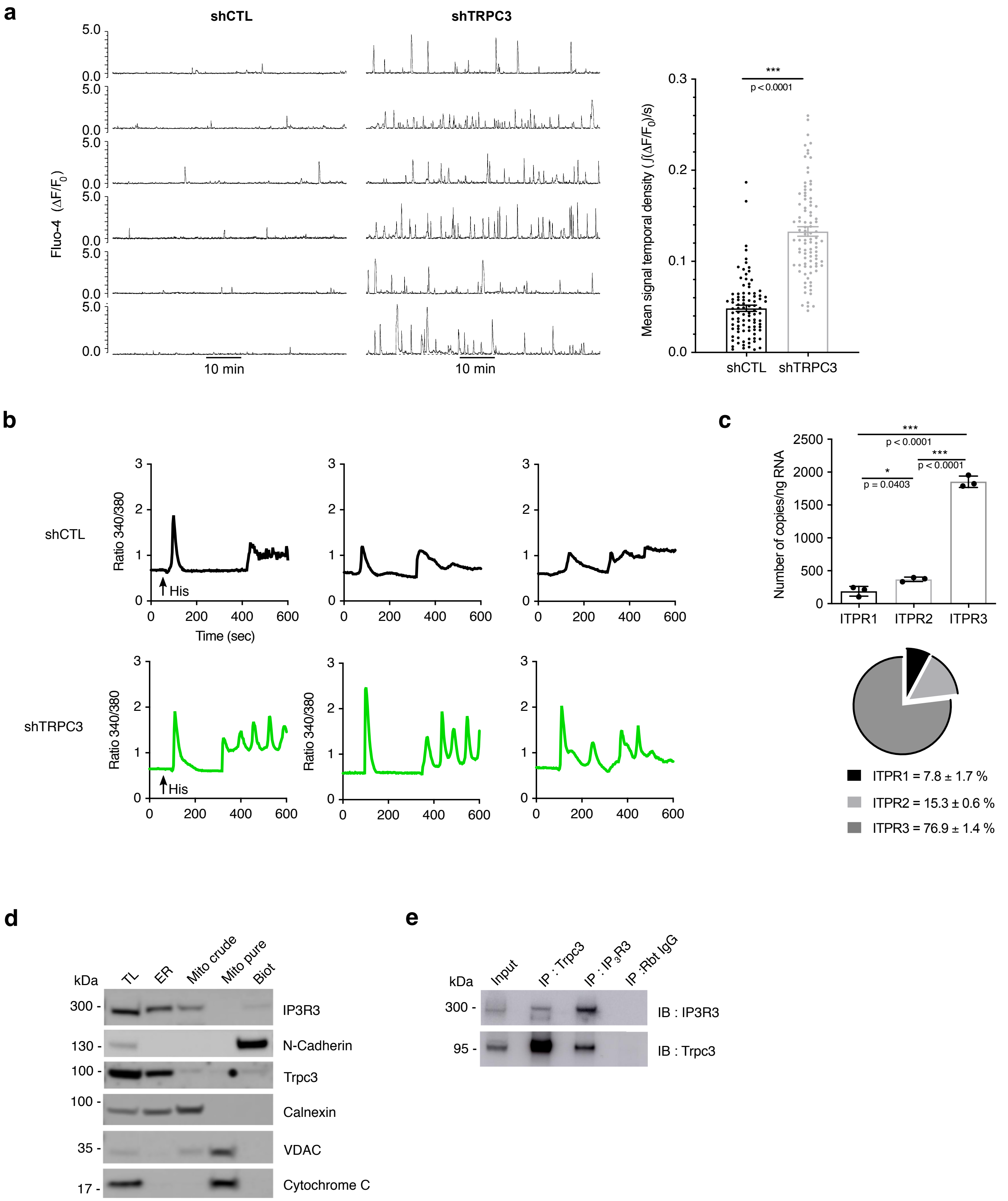

Supplementary Figure 3

(a) Representative traces of spontaneous $[Ca^{2+}]_{cyto}$ oscillations reported by confocal time-series imaging (at 1Hz) of Fluo-4 fluorescence in shCTL (left) or shTRPC3 (middle) MRC5 cells. The bar diagram plot (right) compares corresponding mean signal temporal densities, calculated as signal mass ($\int (\Delta F/F_0)$) per second, in shCTL (n=93) and shTRPC3 (n=97) fibroblasts. Data are presented as mean ± S.E.M. $p< 0.00$, Student's t test, two-sided.

(b) Cytosolic Ca^{2+} imaging (Fura-2) was performed on HPrFs 10 days after induction of either shCTL (top: black) or shTRPC3 (bottom: green). Individual cell time courses of fluorescence ratio with excitation wavelengths 340/380 nm. Note that shTRPC3-transduced cells are prone to cytosolic Ca^{2+} oscillations.

(c) IP_3Rs RNA amount measured with absolute qRT-PCR. The bar diagram plot (left) shows the number of copies/ng total RNA (mean ± S.E.M.; n = 3). ***P<0.001 (Student's t test, two-sided). The pie chart (right) illustrates relative expression of the genes encoding the three IP_3R subtypes.

(d) Immunoblot analysis of the endogenous Trpc3 localization in subcellular fractions obtained from wild-type MRC5. Subcellular origin of the membrane fractions was confirmed with: IP_3R and Calnexin expression (ER), biotinylation and N-Cadherin expression (plasma membrane), (VDAC and Cytochrome C expression (mitochondria). Images are representative of at least three replicates.

(e) Co-immunoprecipitation of TRPC3 and IP_3R3 in MRC5 cells: western blot using anti-TRPC3 and anti-IP_3R3 Ab. Rabbit immunoglobulin G (Rbt IgG) was used as control. Image is representative of one out of three independent biological experiments.

Supplementary Fig. 4

a

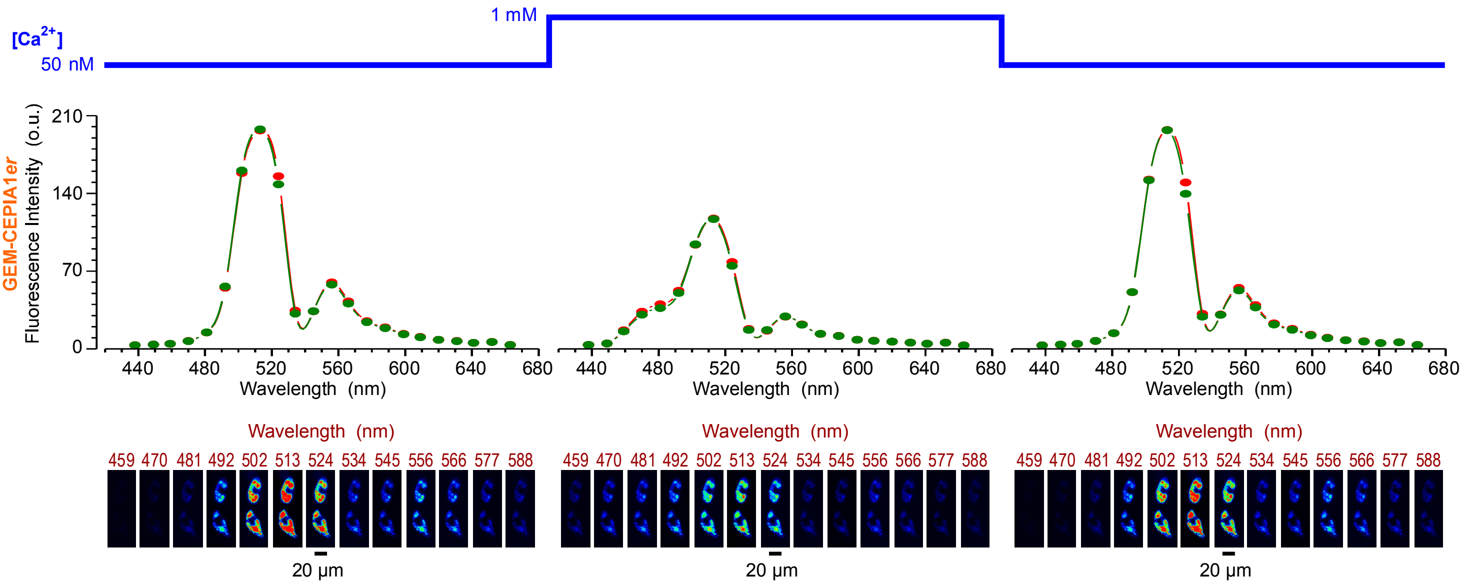

b

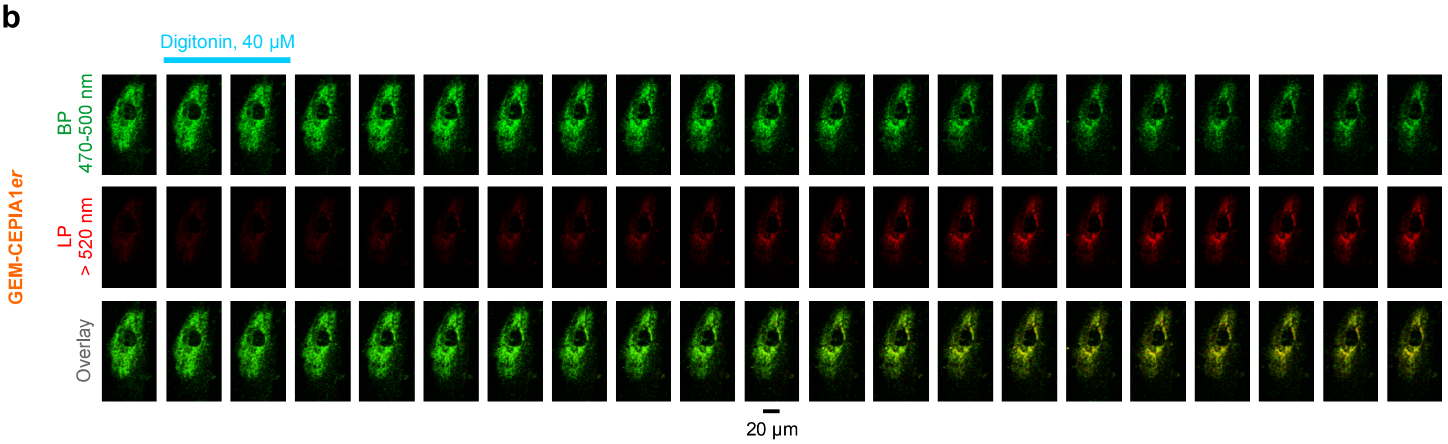

c

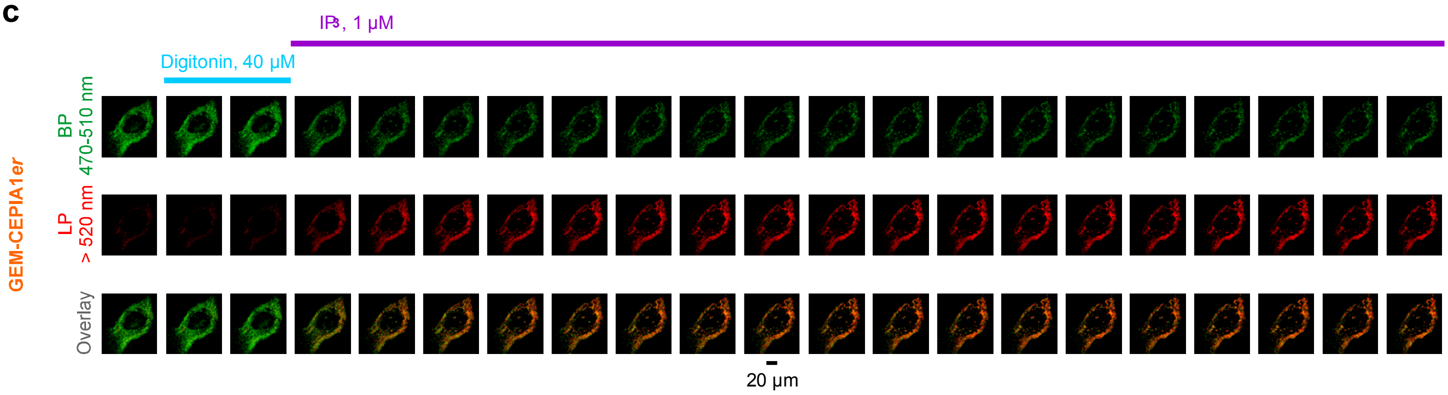

d

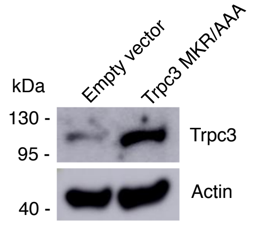

e

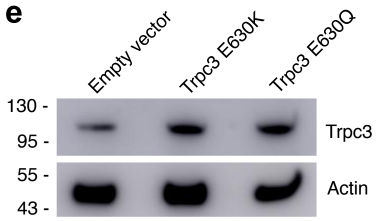

Supplementary Figure 4

(a) To verify optical channel setting for ratiometric confocal $[Ca^{2+}]_{ER}$ imaging, the fluorescence emission spectra of GEM-CEPIA1$_{er}$ expressed in fibroblasts were measured after cell permeabilization (with 40 μM Digitonin) and incubation in a solution with $[Ca^{2+}]$ buffered (EGTA) to either 50 nM or 1 mM (as indicated: top, blue line). Fluorescence was excited by 405 nm line of blue diode laser and captured by META unit of LSM 510 META confocal workstation. The galleries below the plots show rainbow-coded images of GEM-CEPIA1$_{er}$ fluorescence at different wavelength, respectively.

(b) The galleries show the images of GEM-CEPIA1$_{er}$ fluorescence captured at wavelengths 470-500 nm (top), > 520 nm (middle) and their overlay (bottom) during time-laps imaging comprising cell permeabilization (with 40 μM digitonin) in ATP-free, Mg^{2+}-free solution with $[Ca^{2+}]$ buffered to 50 nM, as indicated.

(c) The galleries show the images of GEM-CEPIA1$_{er}$ fluorescence captured as described in (B) except that cell permeabilization was followed by 1 μM IP3 application, as indicated.

(d-e) Immunoblot showing Trpc3 CIRB mutant “MKR/AAA” or pore mutant “E630K and E630Q” overexpression in MRC5 cells. Images are representative of at least three replicates.

Supplementary Fig. 5

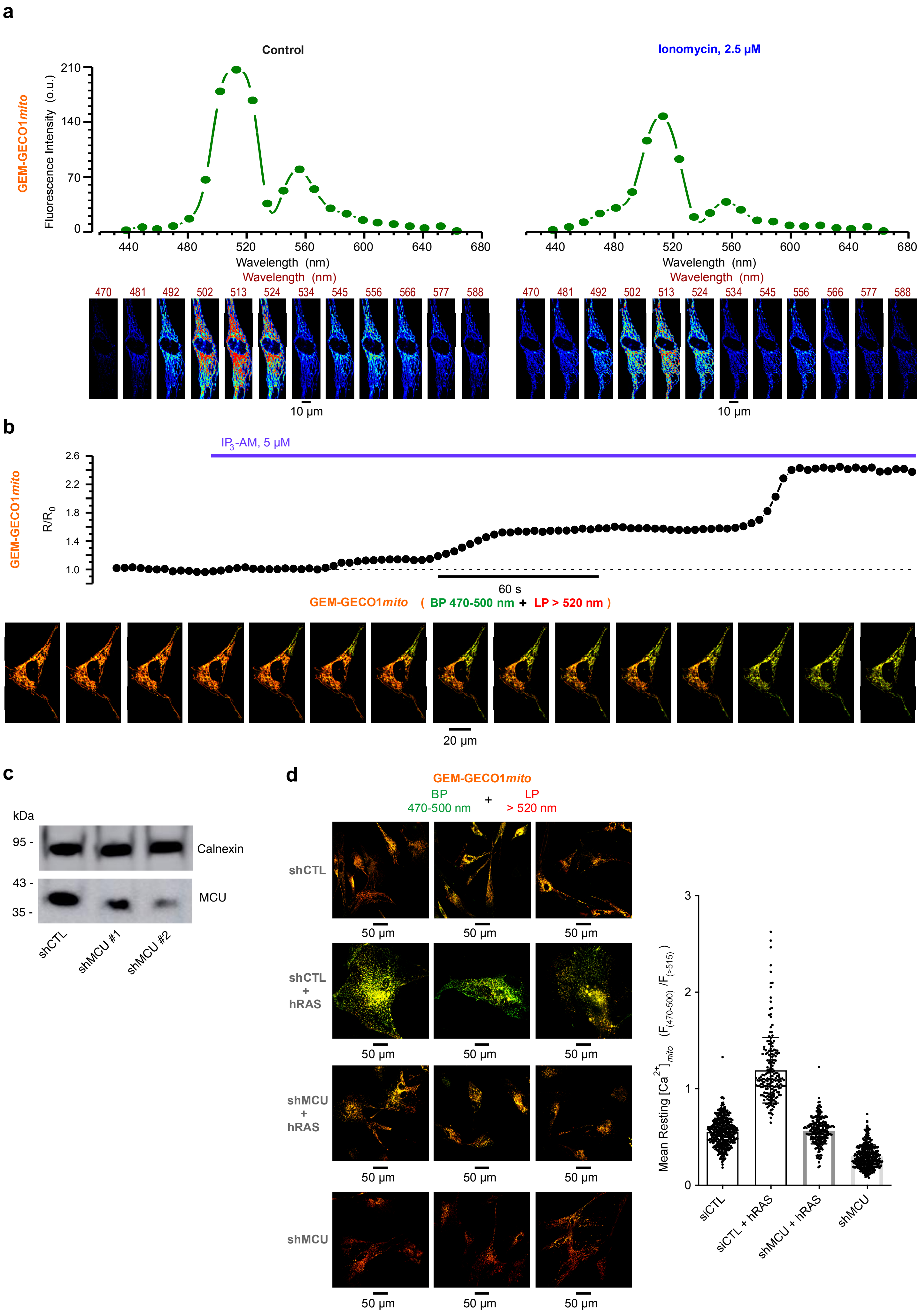

Supplementary Figure 5

(a) To verify optical channel setting for ratiometric confocal $[Ca^{2+}]_{mito}$ imaging, the fluorescence emission spectra of GEM-GECO1*mito* expressed in MRC5 cells were measured in control and after application of 2.5 μM ionomycin, as indicated. Fluorescence was excited by 405 nm line of blue diode laser and captured by META unit of LSM 510 META confocal workstation. The galleries below the plots show rainbow-coded images of GEM-GECO1*mito* fluorescence at different wavelength, respectively.

(b) IP_3-induced Ca^{2+} release triggers mitochondrial Ca^{2+} uptake. The plot (top) shows the temporal profile of the GEM-GECO1*mito* self-normalized fluorescence ratio (R/R_0; where $R=F_{470\text{-}500}/F_{>520}$ at any given time, while R_0 is R averaged before IP_3-AM application), as indicated. The gallery (bottom) shows every 7th image (overlay of 2 channels: BP 470-500 nm and LP > 520 nm) captured during the period corresponding to the trace (top). Note Ca^{2+} wave propagating across the mitochondrial tree.

(c) MCU protein expression upon silencing with shMCU-expressing lentiviral vectors. Western blot images are representative of three independent experiments.

(d) Elevation of mitochondrial Ca^{2+} load upon hRAS overexpression is prevented by mitochondrial Ca^{2+} uniporter (MCU) downregulation. The gallery (left) compares confocal images (overlay of 2 channels: BP 470-500 nm plus LP > 520 nm) of GEM-GECO1*mito* fluorescence from MRC5 treated with control shRNA (shCTL), the shCTL – treated MRC5 overexpressing hRAS (shCTL + hRAS), the hRAS-overexpressing MRC5 treated with shMCU (shMCU + hRAS) or the MRC5 treated with shMCU only (shMCU). The images of 3 representative fields of view per condition are shown. The bar diagram plot (right) compares mean resting $[Ca^{2+}]_{mito}$ (estimated as $F_{470\text{-}500}/F_{>520}$) in control fibroblasts (shCTL: n = 386) with that in the fibroblasts with H-RAS – induced senescence (shCTL + hRAS: n = 197) and the shMCU – treated fibroblasts overexpressing hRAS (shMCU + hRAS: n = 221) or not (shMCU: n = 359). $^{***}P<0.001$ relative to shCTL, unless pointed otherwise. Data are presented as mean ± S.E.M. one-way ANOVA, Tukey's multiple comparisons test.

Supplementary Fig. 6

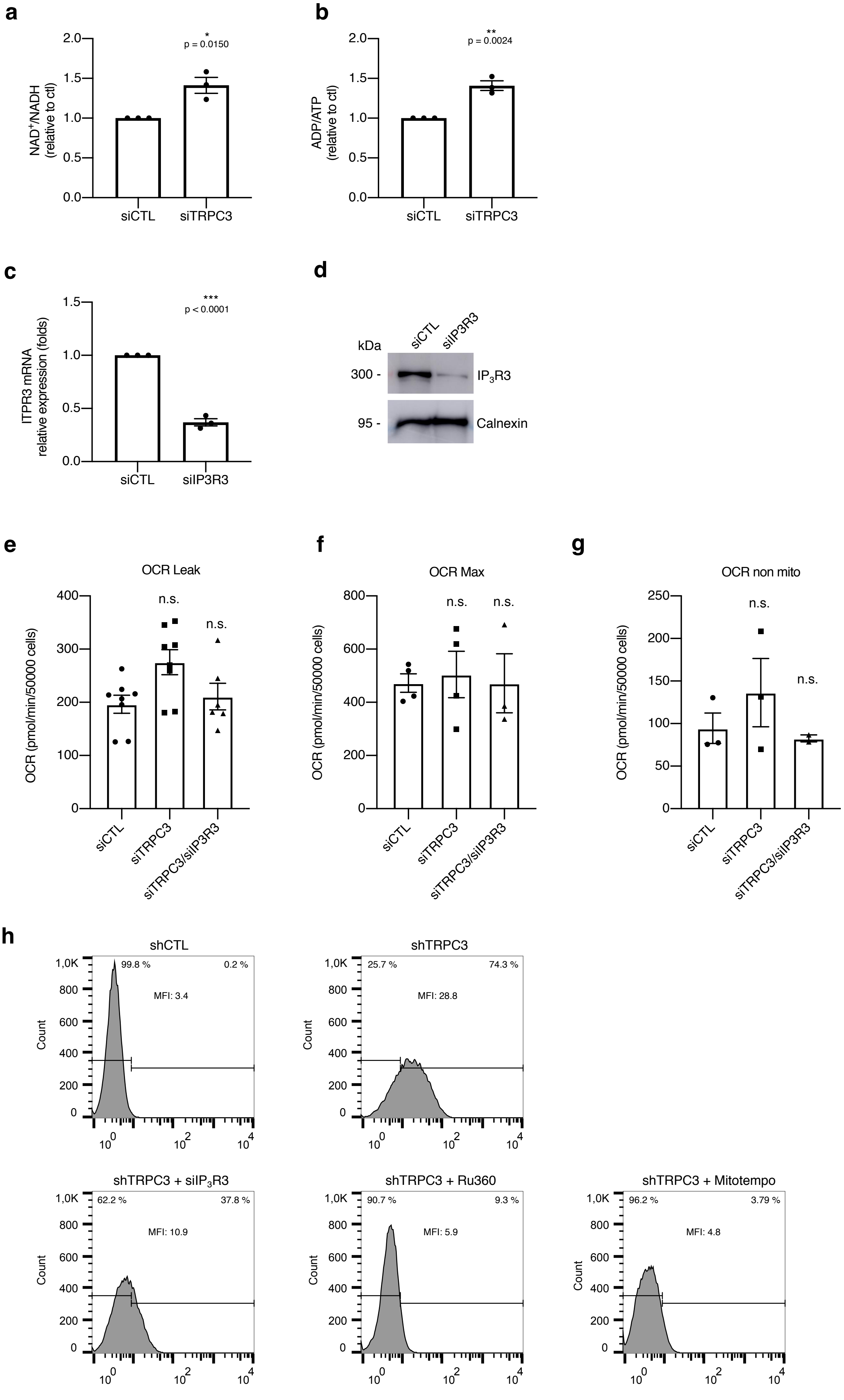

Supplementary Figure 6

(a-b) Bar diagrams showing NAD^+/NADH (A) and ADP/ATP (B) ratios in HPrFs treated either with siCTL or siTRPC3 for 10 days. $^{**}P$<0.01, $^{*}P$<0.05 (Student t test, two-sided). n=3 biological independent experiments. Data are presented as mean ± S.E.M.

(c-d) IP_3R3 mRNA (C) and protein (D) levels 3 days after treatment of MRC5 with either siCTL or $siIP_3R3$ RNAs. Data are expressed as mean ± S.E.M. (n=3 biological independent experiments). Western blot image is representative of three independent experiments. *** *P*<0.001 (Student's t test).

(e-g) States of mitochondrial respiration as shown in Fig. 7C are "leak" (E) for respiration after oligomycin exposure, "Max" (F) for maximal OCR after FCCP exposure and "Non mito" (G) for non-mitochondrial respiration. Mean ± SD; n=4, statistical analyses were performed by one-way ANOVA with a 95% interval of confidence followed by Tukey's post-test (n.s. = not significant).

(h) Flow cytometry histograms showing the distributions of the DDAOG fluorescence intensities reflecting the SA-B-Gal activity in fibroblasts transduced with shCTL or shTRPC3 and in fibroblasts transduced with shTRPC3 and treated either with $siIP_3R3$ (10 nM), the MCU inhibitor Ru360 (5 µM) or the mitochondrial ROS scavenger MitoTempo (10 nM). Corresponding mean fluorescence intensities (MFI) are indicated on the plots. Data are representative of three independent experiments, n = 10.000 for each condition.

Supplementary Fig. 7

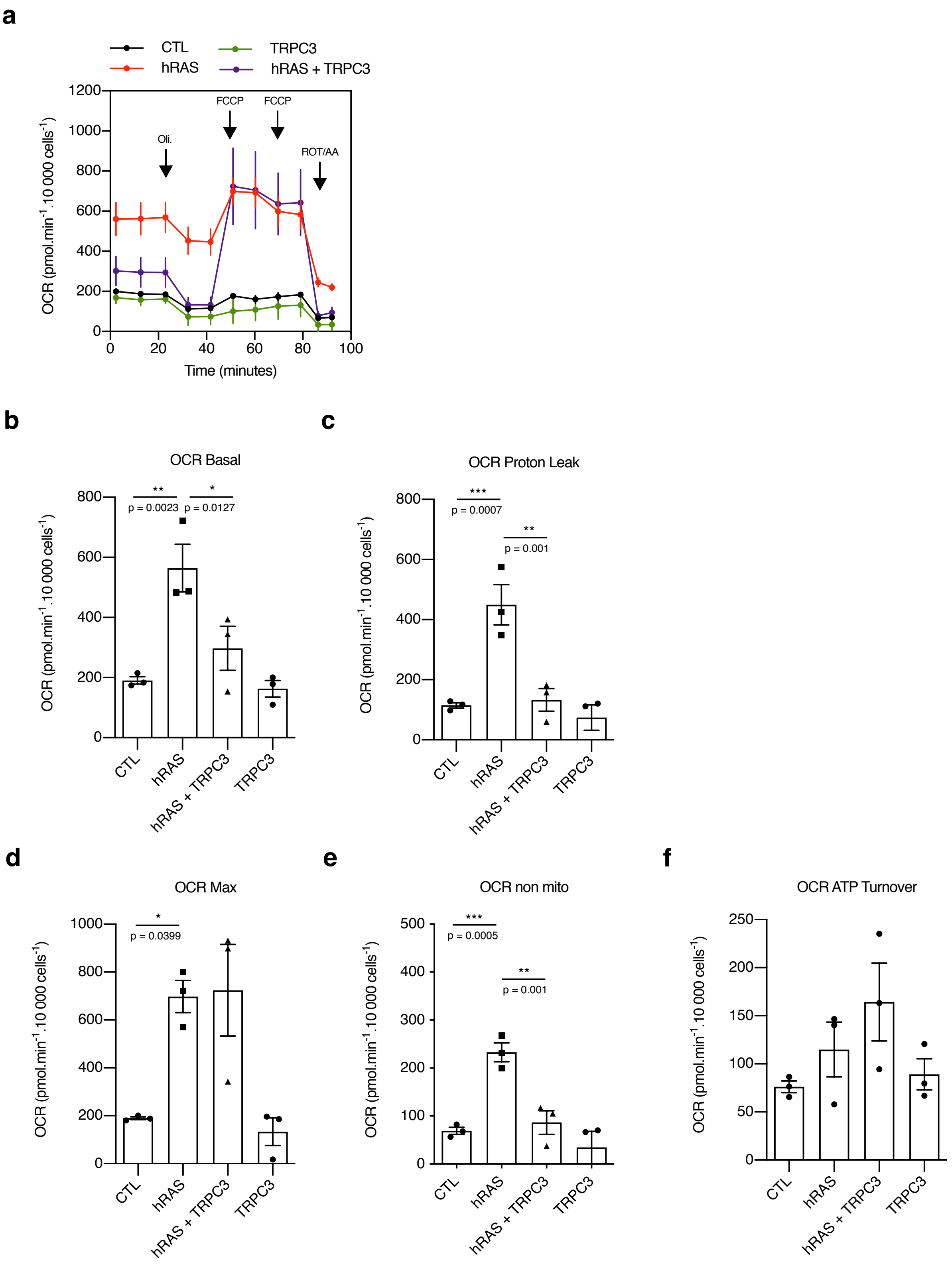

Supplementary Figure 7

(a) Oxygen consumption rate (OCR in pmol $O_2.min^{-1}.50000\ cells^{-1}$) measured using the Seahorse XFe24 technology (mean+/-SD; n=4) on ER:hRAS-MRC5 cells (bearing doxycycline-inducible TRPC3) in which senescence was induced by 4-OHT treatment over 10 days with or without addition of Doxycylin to induce TRPC3 overexpression. Inhibitors have been injected as indicated by arrows: Oligomycin A (Oli.), carbonyl cyanide-4-(trifluoromethoxy)phenylhydrazone (FCCP), and rotenone and antimycin A (Rot/AA). Mean ± SEM; n=4.

(b-f) States of mitochondrial respiration are “basal” (**b**) for basal respiration, “leak” **(c)** for respiration after oligomycin exposure, “Max” **(d)** for maximal OCR after FCCP exposure, “Non mito” **(e)** for non-mitochondrial respiration and “ATP Turnover” (**f**) for ATP-linked OCR (basal measurement minus oligomycin response). Mean ± SD; n=4 (one-way ANOVA followed by Tukeys’s post-test *P< 0.05). Statistical analyses were performed by one-way ANOVA with a 95% interval of confidence followed by Tukey’s post-test. *P< 0.05).

Supplementary Fig. 8

a

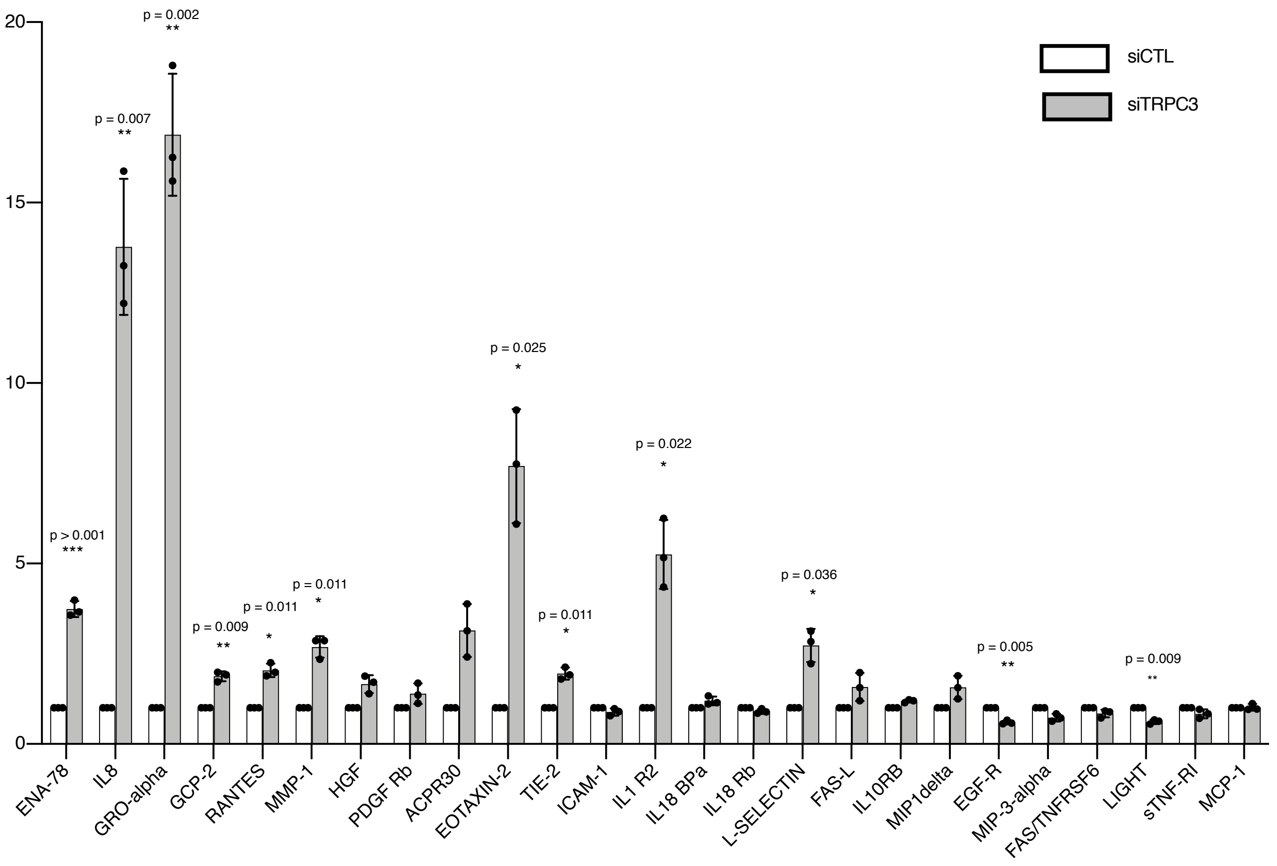

b

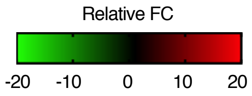

	Fold change for each replicate					
	hRAS vs CTL			hRAS + TRPC3 vs hRAS		
	1	2	3	1	2	3
IL-6	8.2	6.2	6.4	-2.6	-2.7	-1.9
IL-8	164.3	143.7	129.6	-3.4	-4.1	-3.3
IL-11	149.4	98.9	154.0	-13.5	-5.3	-12.4
PAI-1	5.2	5.2	3.2	-5.4	-5.7	-2.7
CXCL1	10.0	6.2	9.2	-2.7	-3.1	-5.2
GM-CSF	398.3	330.0	325.9	-8.2	-4.0	-4.8
CCL5 (RANTES)	4.6	2.7	5.2	-2.2	-2.1	-3.0
TIMP-1	11.1	10.4	8.0	-2.4	-1.7	-2.6
TNFR-I	26.3	67.0	45.6	-6.0	-7.2	-5.3
ICAM-1	8.2	7.2	9.5	-1.1	-0.8	-1.2

Supplementary Figure 8

(a) qRT-PCR showing RNA levels for the SASP components secreted upon TRPC3 KD (please see Fig. 8A). Mean values of the fold changes relative to shCTL are presented in the histogram. Mean ± SEM; n = 3. $^{**}P<0.01$, $^{*}P<0.05$ (Multiple t test, two-sided, Holm-Sidak correction for multiple comparisons).

(b) Heatmap of mRNA levels for the indicated SASP components in ER:hRAS-MRC5 cells with or without TRPC3 rescue by Doxycyclin treatment. Mean values of the fold changes relative to controls (n = 3) are presented in red-green colour scale (see scalebar).

Supplementary Fig. 9

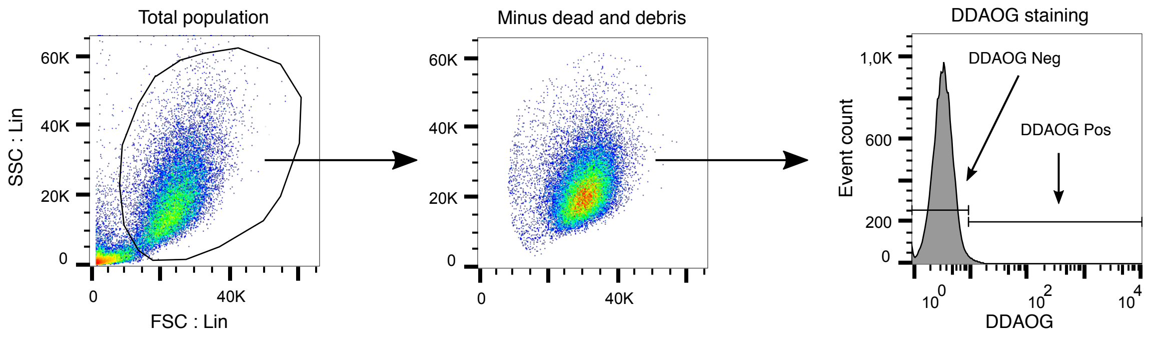

Supplementary Figure 9

FACs gating strategy – representative example. FFSC/SSC gates of the starting cell population were applied to eliminate debris or dead cells. At least 10,000 events were recorded. A representative example of flow cytometry data (dot plots) is provided here.

Supplementary Table 1. List of primers for qPCR

Gene Name	Accession N.	Sequence > 3	
ORAI1	NM_032790	Fw<br>Rv	ATGGTGGCAATGGTGGAG<br>CTGATCATGAGCGCAAACAG
ORAI2	NM_001126340.1	Fw<br>Rv	CCTGTCGTGGCGGAAGCTCT<br>CGGGTACTGGTACTGCGTCT
ORAI3	NM_152288.2	Fw<br>Rv	GGCCAAGCTCAAAGCTTCC<br>CCTGGTGGGTACTCGTGGT
STIM1	NM_003156	Fw<br>Rv	TGTGGAGCTGCCTCAGTATG<br>CTTCAGCACAGTCCCTGTCA
STIM2	NM_001169118.1	Fw<br>Rv	TATGCAAAGGAGGAGGCT<br>ATGCGAACCTGTTCCAATTC
IP_3R1	NM_001099952.2	Fw<br>Rv	CTGATTCACCCACGAAGGTT<br>TGCAAATCAGGTGCTTTCTG
IP_3R2	NM_002223.3	Fw<br>Rv	GCAGGGAAGAAGAGGGACG<br>ACCCCAAGGTGCTGATGAAG
IP_3R3	NM_002224.3	Fw<br>Rv	AACTACCTGGCTGCTGAGGA<br>CGAAAGAGTCGGTTTTCTGC
MCU	NM_138357.3	Fw<br>Rv	CACACAGTTTGGCATTTTGG<br>CTGAGCAATTGCATCCTTGA
VDAC	NM_003374.3	Fw<br>Rv	CGACATGGATTTCGACATTG<br>CCAAACTCTGTCCCGTCATT
NCLX	NM_024959.4	Fw<br>Rv	CCAGGGACCTGGAAGGCTG<br>CGCCTCCGGTGGCCT
CXCL5	NM_002994.5	Fw<br>Rv	TGTGTTGAGAGAGCTGCGTT<br>TCCTTGTTTCCACCGTCCAA
CXCL6	NM_002993.4	Fw<br>Rv	CTGGTCCTGTCTCTGCTGTG<br>TCCGGTCCAGACAAACTTG
CCL5	NM_002985.3	Fw<br>Rv	CTGCCTCCCCATATTCCTCG<br>CACACTTGGCGGTTCTTTCG
MMP1	NM_002421.4	Fw<br>Rv	TCCCAAAATCCTGTCCAGCC<br>CCGGACTTCATCTCTGTCGG
HGF	NM_000601.6	Fw<br>Rv	CAATGCCTCTGGTTCCCCTT<br>CCAGGGCTGACATTTGATGC
PDGFRB	NM_002609.4	Fw<br>Rv	GTTCAAAGACAACCGCACCC<br>GTCTGTTCCCCACTGTCAGG
ACRP30	NM_004797.4	Fw<br>Rv	TCCACTGCAACATTCCTGGG<br>CGCTCTCCTTCCCCATACAC
EOTAXIN2	NM_001371193.1	Fw<br>Rv	CCACCACATCATCCCTACGG<br>TGGCGTCCAGGTTCTTCATG
TIE2	NM_000459.5	Fw<br>Rv	GATGTGATTGGGGAGGGCAA<br>GCGTACTCAATGGCCAGGTA
ICAM1	NM_000201.3	Fw<br>Rv	TGTGACCAGCCCAAGTTGTT<br>AGTCCAGTACACGGTGAGGA
IL1R2	NM_004633.4	Fw<br>Rv	GAGGCATTACAAGCGGGAGT<br>TCTTCTTCTCCTGGGACCGT
IL18BPa	NM_173042.2	Fw<br>Rv	TCGTCACTCTCCTGGTCAGA<br>CTGGCCAGGTCACTTCCAAT

IL18RB	NM_003853.4	Fw<br>Rv	ATGCACAAAGTCCAGCGGTA<br>GCTCTGACTGTCCACGAACT
L-SELECTIN	NM_000655.5	Fw<br>Rv	ATCATGGAACCGACTGCTGG<br>CCCACCCACGTCCATATTCC
FAS-LIGAND	NM_000639.3	Fw<br>Rv	CCCTCCAGGCACAGTTCTTC<br>CTCTTCTTCAGGGGTGGCAG
IL10RB	NM_000628.5	Fw<br>Rv	GATCGGAACAAAGCTGGGGA<br>GTAAACGCACCACAGCAAGG
CCL15	NM_032965.6	Fw<br>Rv	ACTGCTGCACCTCCTACATC<br>CAATCCTGAACTCCCGGACC
EGFR	NM_005228.5	Fw<br>Rv	GTCCGCAAGTGTAAGAAGTGC<br>AGAGGAGGAGTATGTGTGAAGGA
CCL20	NM_004591.3	Fw<br>Rv	ACACAGACCGTATTCTTCATCCT<br>AGACGCACAATATATTTCACCCA
FAS	NM_000043.6	Fw<br>Rv	TGCCCAAGTGACTGACATCA<br>TGTGCAGTCCCTAGCTTTCC
TNFSF14	NM_003807.5	Fw<br>Rv	ATCACCCACGGCCTCTACAA<br>AAAGCCCCGAAGTAAGACCG
TNFRSF1A	NM_001065.4	Fw<br>Rv	TGTGCCTACCCCAGATTGAG<br>GTGGGACTGAAGCTTGGGTT
MCP1	NM_002982.4	Fw<br>Rv	ATGAAAGTCTCTGCCGCCC<br>TTGGGGTCAGCACAGATCTC
IL-11	NM_000641.4	Fw<br>Rv	CTGTGGGGACATGAACTGTG<br>AGGGTCTGGGGAAACTCG
GM-CSF	NM_000758.4	Fw<br>Rv	AGCCACTACAAGCAGCACTG<br>CCAGCAGTCAAAGGGGATGA
PAI-1	NM_001386460.1	Fw<br>Rv	GCCTAATCAGCCCACCATGT<br>TGTACATGGCTGACGTCACC
CXCL-1	NM_001511.4	Fw<br>Rv	TTTCTGAGGAGCCTGCAACA<br>GCACATACATTCCCCTGCCT
GP67	M25420.1	Fw<br>Rv<br>Probe	CTGCAAAAGGACGTGGAAAT<br>CAAATCCTCTTTGCCCACAT<br>6-FAMRA-CGAAAACGTGATTATCGGC-TAMRA
GAPDH	NM_002046.6	Fw<br>Rv	ACCCACTCCTCCACCTTTG3’<br>CTCTTGTGCTCTTGCTGGG3’

Supplementary Table 2. Basal expression levels in untreated cells for the genes showed in Fig.1A

	Ct values (mean) of untreated cells		
**Gene**	**HPrFs**	**CAFs**	**MRC5**
ORAI1	26.61	27.05	26.75
ORAI2	29.65	31.74	26.82
ORAI3	26.91	25.47	27.07
STIM1	25.13	23.65	26.86
STIM2	27.12	26.73	27.71
TRPA1	24.64	24.84	23.68
TRPC1	29.55	26.11	28.26
TRPC3	26.30	26.54	25.94
TRPC4	29.28	35.56	32.95
TRPC5	N/A	N/A	N/A
TRPC6	31.15	31.26	32.70
TRPC7	N/A	N/A	N/A
TRPM1	N/A	N/A	N/A
TRPM2	38.11	37.24	39.50
TRPM3	35.24	34.19	39.81
TRPM4	25.75	25.88	27.14
TRPM5	39.84	38.65	37.97
TRPM6	35.41	37.79	39.89
TRPM7	26.26	26.06	27.97
TRPM8	36.96	36.25	37.80
TRPV1	32.04	30.08	29.71
TRPV2	27.21	27.99	27.59
TRPV3	36.20	34.78	36.72
TRPV4	32.51	31.63	28.71
TRPV5	38.05	37.37	N/A
TRPV6	37.99	35.97	39.56
ITPR1	27.04	27.07	28.35
ITPR2	29.33	29.42	30.26
ITPR3	25.75	26.61	25.78
VDAC	25.78	25.19	28.19
MCU	29.29	28.79	30.61
NCLX1	39.29	38.14	40.93
IL 8	38.05	36.81	35.95

Supplementary table 3. Statistical analysis of qPCR results shown in Fig. 1A

Dunnett's multiple comparisons test	Significant?	Adjusted P Value
ORAI1		
CTL vs. OIS#1	No	0.9559
CTL vs. OXIS#1	No	0.9737
CTL vs. CIS#1	No	0.9689
CTL vs. CIS#2	No	0.4814
ORAI2		
CTL vs. OIS#1	No	0.1160
CTL vs. OXIS#1	No	0.4321
CTL vs. CIS#1	No	0.9269
CTL vs. CIS#2	No	0.1556
ORAI3		
CTL vs. OIS#1	No	0.1329
CTL vs. OXIS#1	No	0.9207
CTL vs. CIS#1	No	0.9788
CTL vs. CIS#2	No	0.9991
STIM1		
CTL vs. OIS#1	No	0.9806
CTL vs. OXIS#1	No	0.9691
CTL vs. CIS#1	No	0.0954
CTL vs. CIS#2	No	0.0945
STIM2		
CTL vs. OIS#1	No	0.9893
CTL vs. OXIS#1	No	0.9973
CTL vs. CIS#1	No	0.3099
CTL vs. CIS#2	No	0.1009
TRPA1		
CTL vs. OIS#1	Yes	0.0103
CTL vs. OXIS#1	No	0.0688
CTL vs. CIS#1	No	0.1198
CTL vs. CIS#2	No	0.9972
TRPC1		
CTL vs. OIS#1	No	0.9098
CTL vs. OXIS#1	No	0.9528
CTL vs. CIS#1	No	0.5093
CTL vs. CIS#2	No	0.1075
TRPC3		
CTL vs. OIS#1	Yes	0.0006
CTL vs. OXIS#1	Yes	0.0024
CTL vs. CIS#1	Yes	0.0050
CTL vs. CIS#2	Yes	0.0118
TRPC4		
CTL vs. OIS#1	No	0.9126
CTL vs. OXIS#1	No	0.6061

CTL vs. CIS#1	No	0.0941
CTL vs. CIS#2	No	0.3582
TRPC5		
CTL vs. OIS#1	No	0.9174
CTL vs. OXIS#1	No	0.9988
CTL vs. CIS#1	No	0.9205
CTL vs. CIS#2	No	0.5482
TRPC6		
CTL vs. OIS#1	No	0.9997
CTL vs. OXIS#1	No	0.4948
CTL vs. CIS#1	No	0.0507
CTL vs. CIS#2	No	0.1190
TRPM1		
CTL vs. OIS#1	Yes	0.0406
CTL vs. OXIS#1	No	0.5475
CTL vs. CIS#1	No	0.5474
CTL vs. CIS#2	No	0.0763
TRPM2		
CTL vs. OIS#1	Yes	0.0036
CTL vs. OXIS#1	No	0.5496
CTL vs. CIS#1	No	0.5515
CTL vs. CIS#2	No	0.9977
TRPM3		
CTL vs. OIS#1	No	0.9985
CTL vs. OXIS#1	No	0.5525
CTL vs. CIS#1	No	0.9965
CTL vs. CIS#2	No	0.9965
TRPM4		
CTL vs. OIS#1	No	0.9422
CTL vs. OXIS#1	No	0.8143
CTL vs. CIS#1	No	0.0541
CTL vs. CIS#2	No	0.0621
TRPM5		
CTL vs. OIS#1	No	0.5422
CTL vs. OXIS#1	No	0.9262
CTL vs. CIS#1	No	0.9499
CTL vs. CIS#2	No	0.9472
TRPM6		
CTL vs. OIS#1	No	0.9565
CTL vs. OXIS#1	No	0.9186
CTL vs. CIS#1	No	0.0832
CTL vs. CIS#2	No	0.7087
TRPM7		
CTL vs. OIS#1	No	0.5452
CTL vs. OXIS#1	No	0.9982
CTL vs. CIS#1	No	0.1127
CTL vs. CIS#2	No	0.3758

TRPM8		
CTL vs. OIS#1	Yes	0.0118
CTL vs. OXIS#1	No	0.7203
CTL vs. CIS#1	No	0.9988
CTL vs. CIS#2	Yes	<0.0001
TRPV1		
CTL vs. OIS#1	No	0.5000
CTL vs. OXIS#1	No	0.9997
CTL vs. CIS#1	No	0.9106
CTL vs. CIS#2	No	0.9137
TRPV2		
CTL vs. OIS#1	No	0.9961
CTL vs. OXIS#1	No	0.9891
CTL vs. CIS#1	No	0.0738
CTL vs. CIS#2	No	0.0798
TRPV3		
CTL vs. OIS#1	No	0.0780
CTL vs. OXIS#1	No	0.9991
CTL vs. CIS#1	No	0.4282
CTL vs. CIS#2	No	0.9989
TRPV4		
CTL vs. OIS#1	No	0.9836
CTL vs. OXIS#1	No	0.9990
CTL vs. CIS#1	No	0.9289
CTL vs. CIS#2	No	0.1580
TRPV5		
CTL vs. OIS#1	No	0.9997
CTL vs. OXIS#1	No	0.9287
CTL vs. CIS#1	No	0.0844
CTL vs. CIS#2	No	0.1602
TRPV6		
CTL vs. OIS#1	No	0.9630
CTL vs. OXIS#1	No	0.5515
CTL vs. CIS#1	No	0.0830
CTL vs. CIS#2	No	0.9861
ITPR1		
CTL vs. OIS#1	No	0.5508
CTL vs. OXIS#1	No	0.4839
CTL vs. CIS#1	Yes	0.0443
CTL vs. CIS#2	No	0.9991
ITPR2		
CTL vs. OIS#1	No	0.4973
CTL vs. OXIS#1	No	0.9663
CTL vs. CIS#1	No	0.0702
CTL vs. CIS#2	No	0.1109
ITPR3		
CTL vs. OIS#1	No	0.9583

CTL vs. OXIS#1	No	0.4863
CTL vs. CIS#1	No	0.9105
CTL vs. CIS#2	No	0.5193
VDAC		
CTL vs. OIS#1	No	0.9999
CTL vs. OXIS#1	No	0.9320
CTL vs. CIS#1	No	0.9757
CTL vs. CIS#2	No	0.9964
MCU		
CTL vs. OIS#1	No	0.8808
CTL vs. OXIS#1	No	0.9766
CTL vs. CIS#1	No	0.9992
CTL vs. CIS#2	No	0.9971
NCLX1		
CTL vs. OIS#1	No	>0.9999
CTL vs. OXIS#1	No	0.9960
CTL vs. CIS#1	No	0.9975
CTL vs. CIS#2	No	0.9885
IL 8		
CTL vs. OIS#1	Yes	<0.0001
CTL vs. OXIS#1	Yes	<0.0001
CTL vs. CIS#1	Yes	<0.0001
CTL vs. CIS#2	Yes	<0.0001

The statistical analysis was performed by 2way Anova with Dunnett's multiple comparison test (vs CTL).
