# Supplementary material for: TRPC3 shapes the ER-mitochondria Ca2+ transfer characterizing tumour-promoting senescence
Source: Nat Commun. 2022 Feb 17;13:956. doi: 10.1038/s41467-022-28597-x (PMC8854551; doi:10.1038/s41467-022-28597-x)
Supplement: Supplementary file 3 — Reporting Summary [file 41467_2022_28597_MOESM3_ESM.pdf]

nature research

Corresponding author(s): Valerio Farfariello, Natalia Prevarskaya

Last updated by author(s): Jan 14, 2021

Reporting Summary

Nature Research wishes to improve the reproducibility of the work that we publish. This form provides structure for consistency and transparency in reporting. For further information on Nature Research policies, see our Editorial Policies and the Editorial Policy Checklist.

Statistics

For all statistical analyses, confirm that the following items are present in the figure legend, table legend, main text, or Methods section.

n/a	Confirmed	
☐	☒	The exact sample size (n) for each experimental group/condition, given as a discrete number and unit of measurement
☒	☐	A statement on whether measurements were taken from distinct samples or whether the same sample was measured repeatedly
☐	☒	The statistical test(s) used AND whether they are one- or two-sided<br>*Only common tests should be described solely by name; describe more complex techniques in the Methods section.*
☐	☒	A description of all covariates tested
☐	☒	A description of any assumptions or corrections, such as tests of normality and adjustment for multiple comparisons
☐	☒	A full description of the statistical parameters including central tendency (e.g. means) or other basic estimates (e.g. regression coefficient) AND variation (e.g. standard deviation) or associated estimates of uncertainty (e.g. confidence intervals)
☐	☒	For null hypothesis testing, the test statistic (e.g. F, t, r) with confidence intervals, effect sizes, degrees of freedom and P value noted<br>*Give P values as exact values whenever suitable.*
☒	☐	For Bayesian analysis, information on the choice of priors and Markov chain Monte Carlo settings
☒	☐	For hierarchical and complex designs, identification of the appropriate level for tests and full reporting of outcomes
☒	☐	Estimates of effect sizes (e.g. Cohen's d, Pearson's r), indicating how they were calculated

Our web collection on statistics for biologists contains articles on many of the points above.

Software and code

Policy information about availability of computer code

Data collection	Flow cytometry data were collected with the Summit Software version 4.3 from Beckman Coulter. In vivo bioluminescence recording was performed with the Living Image software version 4.5 from PerkinElmer. Calcium imaging data were collected with the MetaFluor version 4.5 software. Confocal images and data were collected with the Zen version software from Zeiss Microscopy. Microarray datasets were collected with Oncomine or the R version 3.6.0 software. Primers were designed with the qPrimerDepot version 1.0 software. qRT-PCR data were collected with the CFX Manager version 3.1 software from BioRad.
Data analysis	Flow cytometry data were analysed with the FlowJo version 7.0 software from BD. Statistic analysis were performed with the GraphPad Prism 8 software. Western blot densitometry was performed with the ImageJ/Fiji version 1.53 software. qRT-PCR data analysis were performed with the CFX Manager version 3.1 software from BioRad. GSEA analysis was performed using the GSEA software version 4.1.0 from UC San Diego and Broad Institute.

For manuscripts utilizing custom algorithms or software that are central to the research but not yet described in published literature, software must be made available to editors and reviewers. We strongly encourage code deposition in a community repository (e.g. GitHub). See the Nature Research guidelines for submitting code & software for further information.

Data

Policy information about availability of data

All manuscripts must include a data availability statement. This statement should provide the following information, where applicable:

- Accession codes, unique identifiers, or web links for publicly available datasets
- A list of figures that have associated raw data
- A description of any restrictions on data availability

GSE130727 and GSE35988 datasets used to analyse TRPC3 expression (Supplementary Fig.1 I-L) are available in Gene Expression Omnibus (GEO) (https://www.ncbi.nlm.nih.gov/geo/) and in Oncomine Database (https://www.oncomine.org/) identified as "Grasso Prostate" and "Tamura Prostate". All remaining data will be available from the corresponding author upon reasonable request. The dataset used to calculate gene enrichment (Supplementary Fig. 1M) is available on the Human Cellular Senescence Database (HCSGD) (http://www.bioinfo-xwwang-thu.cn/qdong/HCSGD/). Data relative to the remaining figures are available on the Source File provided with this manuscript.

Field-specific reporting

Please select the one below that is the best fit for your research. If you are not sure, read the appropriate sections before making your selection.

☒ Life sciences ☐ Behavioural & social sciences ☐ Ecological, evolutionary & environmental sciences

For a reference copy of the document with all sections, see nature.com/documents/nr-reporting-summary-flat.pdf

Life sciences study design

All studies must disclose on these points even when the disclosure is negative.

Sample size	For experiments involving calcium imaging and confocal microscopy, sample size was not predetermined. Depending on the number of cells in a single field of view, several technical replicates were produced for each biological replicate in order to give a number of cells sufficient to give statistical significance, if any. For flow cytometry, the count was stopped to at least 10000 events according to the expected elevated frequency of positive events and hence a low coefficient of variation (Roederer M, Cytometry A. 2008 May;73(5):384-5)<br>For each experiment, n=3 was chosen as minimal biological replicate number. For the animal study, minimum sample size was calculated according RB Dell et al. 2002 ILARJ 43 : 207-213 with alpha >/=0.05 and "1-beta"=0.9.
Data exclusions	No data were excluded from the analysis.
Replication	For ethic reasons, the in vivo study was performed once on a group of 10 mice for each condition. All other experiments were done at least in triplicate. All attempts of replication were successful and gave similar results.
Randomization	For in vivo studies, mice were earmarked and randomly assigned, by an independent person, to two groups of 10 subjects receiving PC3-Luc cells along with either shTRPC3 or siCTL prostate fibroblasts.
Blinding	The investigators were not blinded to the groups allocation during the experiments. Blinding was not possible as experimental conditions were evident from data (different morphology of senescent cells or use of concomitant senescence markers).

Reporting for specific materials, systems and methods

We require information from authors about some types of materials, experimental systems and methods used in many studies. Here, indicate whether each material, system or method listed is relevant to your study. If you are not sure if a list item applies to your research, read the appropriate section before selecting a response.

Materials & experimental systems

n/a	Involved in the study	
☐	☒	Antibodies
☐	☒	Eukaryotic cell lines
☒	☐	Palaeontology and archaeology
☐	☒	Animals and other organisms
☒	☐	Human research participants
☒	☐	Clinical data
☒	☐	Dual use research of concern

Methods

n/a	Involved in the study	
☒	☐	ChIP-seq
☐	☒	Flow cytometry
☒	☐	MRI-based neuroimaging

Antibodies

Antibodies used

- Rabbit polyclonal anti-TRPC3; WB dilution: 1/200; IP dilution: 5µg/mg lysate; Alomone Labs Cat# ACC-016
- Mouse monoclonal anti-GAPDH clone 6C5; WB dilution: 1/2000; Abcam Cat# ab8245
- Mouse monoclonal anti-Beta-Actin clone AC-74; WB dilution: 1/2000; Sigma Aldrich Cat# A2228
- Rabbit polyclonal anti-IP3R3; IP dilution: 5µg/mg lysate; Bethyl Laboratories Cat# A302-160A
- Mouse monoclonal anti-IP3R3 clone 2/IP3R-3; IB dilution 1/1000; BDBiosciences Cat# 610313
- Rabbit monoclonal anti-N-Cadherin clone H-63; WB dilution: 1/200; SantaCruz Biotech. Cat# sc-7939
- Mouse monoclonal anti-Calnexin clone C8.B6; WB dilution: 1/2000 Millipore Cat# MAB3126
- Mouse monoclonal anti-VDAC1 clone B-6; WB dilution: 1/200; SantaCruz Biotech. Cat# sc-390996
- Mouse monoclonal anti-Cytocrome C clone A-8; WB dilution: 1/200 SantaCruz Biotech. Cat# sc-13156
- Rabbit monoclonal anti-MCU clone D2Z3B; WB dilution: 1/1000; Cell Signaling Cat# 149975
- Mouse monoclonal anti P53 clone DO-1; WB dilution 1/200; SantaCruz Biotech. Cat# sc-126
- Mouse monoclonal anti PCNA1 clone PC10; WB dilution 1/200; SantaCruz Biotech. Cat# sc-56
- Mouse monoclonal anti Cyclin D1 clone G124-326; WB dilution 1/1000; BD Pharmingen. Cat# 554180
- Goat polyclonal anti p16INK4a / CDKN2A; WB dilution 1 µg/mL; R&D systems Cat# AF5779
- Rat monoclonal anti hRAS clone 342404; WB dilution 5 µg/mL; R&D systems Cat# MAB3429
- IgG from rabbit serum Sigma Aldrich Cat# I-5006; IP dilution: 5µg/mg lysate
- HRP-conjugated goat anti-rabbit IgG; WB dilution 1/50000
- HRP-conjugated goat anti-mouse IgG; WB dilution 1/25000

Validation

- Rabbit polyclonal anti-TRPC3; Alomone Labs Cat# ACC-016 : validated for WB in Human, Mouse and Rat by the manufacturer and by data provided in this manuscript using shTRPC3 or siTRPC3
- Mouse monoclonal anti-GAPDH clone 6C5; Abcam Cat# ab8245 : more than 3600 citations in CiteAb website and validated according to the detected band size
- Mouse monoclonal anti-Beta-Actin clone AC-74; Sigma Aldrich Cat# A2228 : more than 2500 citations in CiteAb website and validated according to the detected band size
- Rabbit polyclonal anti-IP3R3; Bethyl Laboratories Cat# A302-160A : validated by data provided in this manuscript using siIP3R3 and Co-IP
- Mouse monoclonal anti-IP3R3 clone 2/IP3R-3; BDBiosciences Cat# 610313 : validated by data provided in this manuscript using siIP3R3
- Rabbit monoclonal anti-N-Cadherin clone H-63; SantaCruz Biotech. Cat# sc-7939 : validated by data provided in this manuscript by biotinylation assay (enrichment) and according to the detected band size
- Mouse monoclonal anti-Calnexin clone C8.B6; Millipore Cat# MAB3126 : KO-validated by Prior K et al. J Biol Chem. 2016;291:7045-59
- Mouse monoclonal anti-VDAC1 clone B-6; SantaCruz Biotech. Cat# sc-390996 : validated by data provided in this manuscript by cell fractionation assay (enrichment) and according to the detected band size
- Mouse monoclonal anti-Cytocrome C clone A-8; SantaCruz Biotech. Cat# sc-13156 : validated by data provided in this manuscript by cell fractionation assay (enrichment) and according to the detected band size
- Rabbit monoclonal anti-MCU clone D2Z3B; Cell Signaling Cat# 149975 : validated by data provided in this manuscript by shMCU and according to the detected band size
- Mouse monoclonal anti P53 clone DO-1; SantaCruz Biotech. Cat# sc-126 : more than 4800 citations in CiteAb website and validated by data provided in this manuscript with the use of positive controls (senescent cells lysates) and according to the detected band size
- Mouse monoclonal anti PCNA1 clone PC10; SantaCruz Biotech. Cat# sc-56 : more than 2300 citations in CiteAb website, validated by data provided in this manuscript according to the detected band size and accumulation in proliferating cells
- Mouse monoclonal anti Cyclin D1 clone G124-326; BD Pharmingen. Cat# 554180 : validated by data provided in this manuscript according to the detected band size and accumulation in proliferating cells
- Goat polyclonal anti p16INK4a / CDKN2A; R&D systems Cat# AF5779 : validated by data provided in this manuscript according to the detected band size and with the use of positive controls (senescent cells lysates)
- Rat monoclonal anti hRAS clone 342404; R&D systems Cat# MAB3429 : validated by data provided in this manuscript according to the detected band size and with the use of hRAS overexpression
- IgG from rabbit serum Sigma Aldrich Cat# I-5006; IP dilution: 5µg/mg lysate
- HRP-conjugated goat anti-rabbit IgG; WB dilution 1/50000
- HRP-conjugated goat anti-mouse IgG; WB dilution 1/25000

Eukaryotic cell lines

Policy information about cell lines

Cell line source(s)

- H. sapiens: MRC-5 fibroblasts, PD 5-40, ATCC Cat#CCL-171
- H. sapiens: Normal Prostate Fibroblasts, PD 0-15, ScienCell Cat#4410
- H. sapiens: Cancer-associated fibroblasts, PD 0-15, see Vancauwenberghe, E. et al. Activation of mutated TRPA1 ion channel by resveratrol in human prostate cancer associated fibroblasts (CAF). Mol. Carcinog. 56, 1851–1867 (2017)
- H. sapiens: PC3 ATCC CRL-1435
- H. sapiens: PC3-Luc (derived from ATCC CRL-1435)
- H. sapiens: DU145 ATCC HTB-81
- H. sapiens: Human Embrionic Kidney GP-293, Clontech Cat#631458
- H. sapiens: Lenti-X 293T, Horizon Discovery, Clontech Cat# 632180
- S. frugiperda: Sf9, Novagen Cat#71104

Authentication

- MRC-5 fibroblasts, PC3 and DU145 prostate cancer cells were authenticated by ATCC by STR profiling
- Normal Prostate Fibroblasts were not authenticated but confirmed by ScienCell by morphology and immunofluorescent fibronectin staining

	- Cancer-associated fibroblasts were not authenticated but confirmed as published in Mol. Carcinog. 56, 1851–1867 (2017)<br>- Human Embrionic Kidney GP-293 and Lenti-X 293T cells were bought from TakaraBio without further authentication<br>- Sf9 cells were bought from Merck-Millipore without further authentication
Mycoplasma contamination	All cell lines and strains were free from Mycoplasma contamination, as confirmed by DAPI staining and fluorescence microscopy analysis.
Commonly misidentified lines<br>(See ICLAC register)	No commonly misidentified cell lines were used in the study

Animals and other organisms

Policy information about studies involving animals; ARRIVE guidelines recommended for reporting animal research

Laboratory animals	M. musculus: Strain NOD.CB17-Prkdscid/NCrHsd, male, aged 5 weeks
Wild animals	No wild animals were used in the study
Field-collected samples	No field collected samples were used in the study
Ethics oversight	Protocols and sample sizes, determined in order to have enough samples to detect statistical differences, if any, were approved by the Animal Experimentation Ethics Committee CEEA Nord - Pas de Calais n°75 (Permit No. 2017062015327270), and all procedures were conducted in accordance with the French National Chart for Animal Experimentation and the ARRIVE guidelines.

Note that full information on the approval of the study protocol must also be provided in the manuscript.

Flow Cytometry

Plots

Confirm that:

☒ The axis labels state the marker and fluorochrome used (e.g. CD4-FITC).

☒ The axis scales are clearly visible. Include numbers along axes only for bottom left plot of group (a 'group' is an analysis of identical markers).

☒ All plots are contour plots with outliers or pseudocolor plots.

☒ A numerical value for number of cells or percentage (with statistics) is provided.

Methodology

Sample preparation	Were necessary, fibroblasts (MRC5 or Human Prostate Fibroblast) were were incubated with 5 µl of Pacific Blue Annexin-V and 5 ul of 7-AAD for 15 min at +4 °C or with DDAOG for 3 h at 37 °C 5% CO2 before analysis.
Instrument	CYAN AD (Beckman Coulter, USA)
Software	- Summit Software version 4.3 from Beckman Coulter for acquisition<br>- FlowJo v.7.0 from BD for analysis
Cell population abundance	At least 10000 cells were acquired for each sample
Gating strategy	FFSC/SSC gates of the starting cell population were only applied to data shown in Fig. S6H to eliminate debris or dead cells, as shown in the representative figure provided in the supplementary information. Boundaries between "positive" and "negative" staining cell populations are defined each time by the control sample (shCTL or siCTL).

☒ Tick this box to confirm that a figure exemplifying the gating strategy is provided in the Supplementary Information.
